# Hypertrophic cardiomyopathy management: a systematic review of the clinical practice guidelines and recommendations

**DOI:** 10.1093/ehjqcco/qcae117

**Published:** 2025-01-02

**Authors:** Mihir M Sanghvi, Eamon Dhall, C Anwar A. Chahal, Constantinos O'Mahony, Saidi A Mohiddin, Konstantinos Savvatis, Fabrizio Ricci, Patricia B Munroe, Steffen E Petersen, Nay Aung, Mohammed Y Khanji

**Affiliations:** William Harvey Research Institute, Queen Mary University of London, Charterhouse Square, London EC1M 6BQ, UK; NIHR Barts Biomedical Research Centre, Queen Mary University of London, Charterhouse Square, London EC1M 6BQ, UK; Barts Heart Centre, Barts Health NHS Trust, West Smithfield, London EC1A 7BE, UK; Newham University Hospital, Barts Health NHS Trust, Glen Road, London E13 8SL, UK; William Harvey Research Institute, Queen Mary University of London, Charterhouse Square, London EC1M 6BQ, UK; Barts Heart Centre, Barts Health NHS Trust, West Smithfield, London EC1A 7BE, UK; Center for Inherited Cardiovascular Diseases, Department of Cardiology, WellSpan Health, 30 Monument Road, York, PA 17403, USA; Department of Cardiovascular Medicine, Mayo Clinic, 200 First Street SW, Rochester, MN 55905, USA; Barts Heart Centre, Barts Health NHS Trust, West Smithfield, London EC1A 7BE, UK; UCL Institute of Cardiovascular Science, University College London, Gower Street, London WC1E 6BT, UK; NIHR University College London Hospitals Biomedical Research Centre, University College London, Gower Street, London WC1E 6BT, UK; William Harvey Research Institute, Queen Mary University of London, Charterhouse Square, London EC1M 6BQ, UK; NIHR Barts Biomedical Research Centre, Queen Mary University of London, Charterhouse Square, London EC1M 6BQ, UK; Barts Heart Centre, Barts Health NHS Trust, West Smithfield, London EC1A 7BE, UK; William Harvey Research Institute, Queen Mary University of London, Charterhouse Square, London EC1M 6BQ, UK; NIHR Barts Biomedical Research Centre, Queen Mary University of London, Charterhouse Square, London EC1M 6BQ, UK; Barts Heart Centre, Barts Health NHS Trust, West Smithfield, London EC1A 7BE, UK; UCL Institute of Cardiovascular Science, University College London, Gower Street, London WC1E 6BT, UK; NIHR University College London Hospitals Biomedical Research Centre, University College London, Gower Street, London WC1E 6BT, UK; Department of Neuroscience, Imaging and Clinical Sciences, “G. d'Annunzio” University of Chieti-Pescara, Via dei Vestini 33, 66100 Chieti, Italy; University Cardiology Division, SS Annunziata Polyclinic University Hospital, Via dei Vestini 5, 66100 Chieti, Italy; Institute for Advanced Biomedical Technologies, “G. d'Annunzio” University of Chieti-Pescara, 66100 Chieti, Italy; William Harvey Research Institute, Queen Mary University of London, Charterhouse Square, London EC1M 6BQ, UK; NIHR Barts Biomedical Research Centre, Queen Mary University of London, Charterhouse Square, London EC1M 6BQ, UK; William Harvey Research Institute, Queen Mary University of London, Charterhouse Square, London EC1M 6BQ, UK; NIHR Barts Biomedical Research Centre, Queen Mary University of London, Charterhouse Square, London EC1M 6BQ, UK; Barts Heart Centre, Barts Health NHS Trust, West Smithfield, London EC1A 7BE, UK; William Harvey Research Institute, Queen Mary University of London, Charterhouse Square, London EC1M 6BQ, UK; NIHR Barts Biomedical Research Centre, Queen Mary University of London, Charterhouse Square, London EC1M 6BQ, UK; Barts Heart Centre, Barts Health NHS Trust, West Smithfield, London EC1A 7BE, UK; William Harvey Research Institute, Queen Mary University of London, Charterhouse Square, London EC1M 6BQ, UK; NIHR Barts Biomedical Research Centre, Queen Mary University of London, Charterhouse Square, London EC1M 6BQ, UK; Barts Heart Centre, Barts Health NHS Trust, West Smithfield, London EC1A 7BE, UK; Newham University Hospital, Barts Health NHS Trust, Glen Road, London E13 8SL, UK

**Keywords:** Hypertrophic cardiomyopathy, Cardiomyopathies, Systematic review, Practice guidelines, Risk stratification

## Abstract

**Aims:**

In light of recent advances in imaging techniques, molecular understanding and therapeutic options in hypertrophic cardiomyopathy (HCM), we performed a systematic review of current guidelines for the diagnosis and management of HCM in order to identify consensus and discrepant areas in the clinical practice guidelines.

**Methods and results:**

We systematically reviewed the English language guidelines and recommendations for the management of HCM in adults. MEDLINE and EMBASE databases were searched for guidelines published in the last 10 years. Following a systematic search, three guidelines on the diagnosis and management of HCM were identified, all of which were robustly developed (AGREE rigour of development score ≥50%). These guidelines were authored by the major European (European Society of Cardiology; 2023), American (American Heart Association /American College of Cardiology/American Medical Society for Sports Medicine /Heart Rhythm Society/Pediatric and Congenital Electrophysiology Society/Society for Cardiovascular Magnetic Resonance; 2024), and Japanese [Japanese Circulation Society (JCS)/Japanese Heart Failure Society (JHFS); 2018] cardiovascular societies. There was broad consensus on echocardiographic recommendations, the medical and invasive management of HCM, the application of genetic testing and family screening, and exercise and reproductive recommendations in HCM. There were areas of variability in the definition and diagnostic criteria for HCM, cardiovascular magnetic resonance imaging recommendations, and assessment of sudden cardiac death (SCD) risk and prevention strategies. Due to the JCS/JHFS guidelines being older, there are no recommendations on the use of cardiac myosin ATPase inhibitors.

**Conclusion:**

Contemporary guidelines for HCM achieve consensus across a broad range of criteria and recommendations concerning diagnosis and management. However, variations in the approach towards risk assessment for SCD exist between the guidelines. There are also more subtle differences concerning diagnostic criteria and the utility of late gadolinium enhancement for risk stratification, which will likely evolve as the evidence-base broadens.

Key learning points **What is already known**

- There have been several guidelines on the diagnosis and management of hypertrophic cardiomyopathy in recent years, particularly as our understanding of the molecular basis of the disease, and the evidence base around risk stratification using advanced imaging techniques and novel therapeutic options has grown.

**What this study adds**

- This is the first systematic review of the current international guidelines on the diagnosis and management of hypertrophic cardiomyopathy to assess areas of consensus and discrepant practice. It is hoped that this will aid clinical decision-making and highlight areas for future research.

## Introduction

Hypertrophic cardiomyopathy (HCM) is characterized by the presence of increased left ventricular (LV) wall thickness or mass (hypertrophy).^1^ It is a genetically determined heart muscle disease most often caused by mutations in sarcomeric genes which encode proteins forming the contractile apparatus of the heart, although many features of the HCM phenotype may not be attributable to sarcomere mutations.^2^ HCM is notable for the heterogeneous spectrum of its phenotypic expression, clinical course, and the pattern and extent of LV hypertrophy, and other features such as papillary muscle and mitral valve abnormalities. It is associated with an increased risk of sudden (arrhythmic) cardiac death, atrial fibrillation, thromboembolic disease, and progressive heart failure. HCM has an estimated prevalence of 1 in 500 individuals.

With advances in imaging techniques, improved molecular understanding and novel therapeutic options in HCM over recent years, there have been updates to clinical practice guideline recommendations. We performed a systematic review of current guidelines for the diagnosis and management of HCM to identify similarities and differences in the guidelines and support clinicians in decision-making and highlight potential gaps that may be overcome with further research or included in future guidelines.

## Methods

### Data sources and searches

We conducted a systematic review of English language guidelines and recommendations for the management of HCM in adults. MEDLINE and EMBASE databases were searched for guidelines published in the last 10 years on 20th May 2024. We also searched websites of organizations relevant to guideline development (Supplementary material online, Table S1). This systematic review was planned, conducted, and reported in agreement with the Preferred Reporting Items for Systematic Reviews and Meta-Analyses (PRISMA) recommendations.^3^

### Study selection

A comprehensive search strategy (Supplementary material online) was constructed to identify potentially relevant literature. Key search terms included ‘cardiomyopathy’, ‘hypertrophic cardiomyopathy’, ‘HCM’, ‘HOCM’, ‘guideline*’, and ‘recommendation*’. Included articles were those published by professional societies or organizations which made specific recommendations for the diagnosis and management of HCM and met the Institute of Medicine's definition of a guideline. Guidelines on cardiomyopathies with HCM making part of the guideline were considered. Consensus statements were excluded and only the most recent guideline from an organization/society was included in the analysis.

### Data extraction and quality assessment

Titles and abstracts were assessed by two independent reviewers (M.M.S. and E.D.) using the software tool Rayyan.^4^ Articles were excluded if both reviewers agreed they were ineligible. Discrepancies were resolved by consensus after discussion. Both reviewers performed the final selection for full data extraction.

The Appraisal of Guidelines for Research and Evaluation (AGREE) II instrument was used to determine the rigour of development for each guideline. Two reviewers (M.M.S. and E.D.) independently rated the items, as per the instructions of the AGREE II tool.^5^ The average rigour scores were obtained by expressing the sum of the individual scores as a percentage of the maximum possible score with reproducibility of grading assessed by interclass correlation. Editorial independence from the funding body, external funding, and disclosure of relationships with industry by individual guideline group members were also assessed. Two reviewers (M.M.S. and E.D.) extracted all relevant recommendations from the guidelines that had an AGREE II score ≥50% (Supplementary material online).

## Results

**Figure 1:**
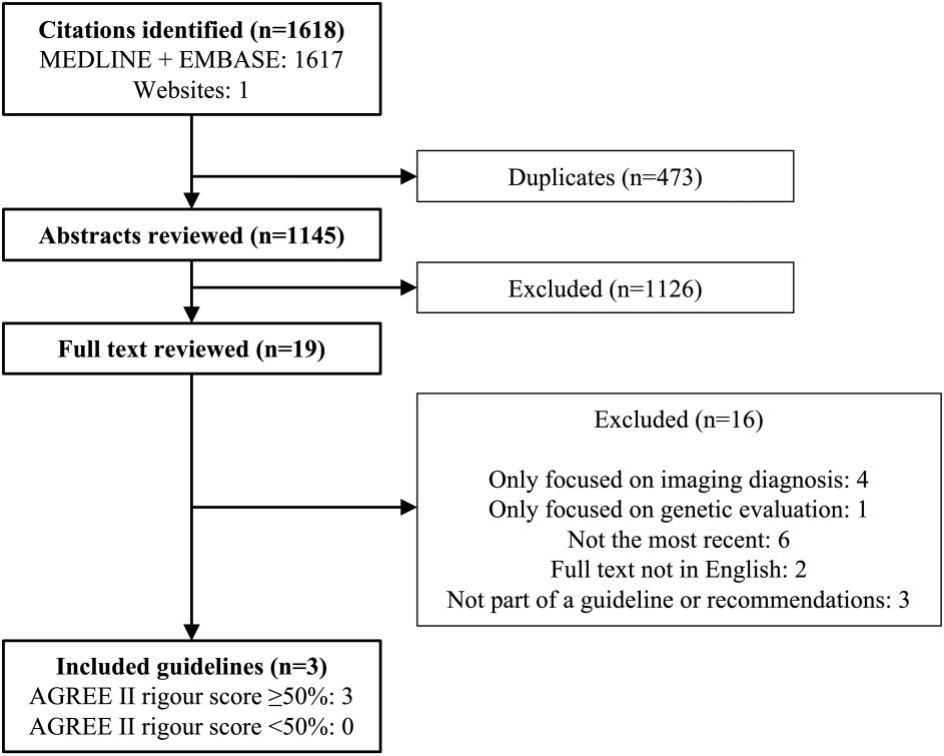
Summary of the guideline search and review process. The number of guidelines at each step is indicated. AGREE II, Appraisal of Guidelines for Research and Evaluation II.

The search strategy, following deduplication, retrieved 1145 manuscripts. Following title and abstract screening, 19 articles were taken forward to full-text screening. Following full-text review, a total of three guidelines on the management of cardiomyopathies or the management of HCM were deemed eligible. Reproducibility of the two reviewers’ rigour scores was good, with an interclass correlation of 0.89. All three documents achieved a rigour score of ≥50%, and were taken forward as the basis of this analysis. A summary of the screening process and exclusions is detailed in *Figure 1*.

The guidelines were from the following societies or organizations: European Society of Cardiology (ESC), American Heart Association (AHA), American College of Cardiology (ACC), American Medical Society for Sports Medicine (AMSSM), Heart Rhythm Society (HRS), Pediatric and Congenital Electrophysiology Society (PACES), Society for Cardiovascular Magnetic Resonance (SCMR), Japanese Circulation Society (JCS), Japanese Heart Failure Society (JHFS). These three guidelines were published in 2023,^6^ 2024,^7^ and 2018,^8^ respectively. A summary of the guideline recommendations is provided in *Table 1*.

**Table 1:** Summary of guideline recommendations for the diagnosis and management of hypertrophic cardiomyopathy

	2023 ESC Guidelines for the management of cardiomyopathies^6^	2024 AHA/ACC/AMSSM/HRS/PACES/SCMR Guideline for the management of hypertrophic cardiomyopathy^7^	JCS/JHFS 2018 Guideline on the diagnosis and treatment of cardiomyopathies^8^
Document type	Clinical practice guideline	Clinical practice guideline	Clinical practice guideline
Year	2023	2024	2018
AGREE II rigour score (%)	82	68	52
Conflict of interest	EI^a^, SCI^b^	EI^a^, SCI^b^	EI^a^, SCI^b^
Method used to evaluate evidence	Not stated	Systematic review	Not stated
Method used to formulate recommendations	Formal consensus	Formal consensus	Formal consensus
Definition of HCM	The presence of increased LV wall thickness (with or without RV hypertrophy) or mass that is not solely explained by abnormal loading conditions.	A disease state in which morphologic expression is confined solely to the heart. It is characterized predominantly by LVH in the absence of another cardiac, systemic, or metabolic disease capable of producing the magnitude of hypertrophy evident in a given patient and for which a disease-causing sarcomere (or sarcomere-related) variant is identified, or genetic aetiology remains unresolved.	A disease state in which a pathogenic mutation has bene identified mainly in genes coding for sarcomere proteins, or in which LVH associated with other cardiac diseases, including storage, infiltrative, or systemic disease, has been excluded.
Diagnostic criteria	HCM is defined as an LV wall thickness of ≥15 mm in any myocardial segment that is not explained solely by loading conditions. Lesser degrees of wall thickening (13–14 mm) require evaluation of other features including family history, genetic findings, and ECG abnormalities. A diagnosis of HCM in adult first-degree relatives of patients with unequivocal disease is based on the presence of LV wall thickness ≥13 mm	A maximal end-diastolic wall thickness of ≥15 mm anywhere in the left ventricle, in the absence of another cause of hypertrophy in adults. More limited hypertrophy (13–14 mm) can be diagnostic when present in family members of a patient with HCM or in conjunction with a positive genetic test identifying a pathogenic or likely pathogenic variant often in a sarcomere gene.	A maximum LV wall thickness ≥15 mm Maximum LV wall thickness ≥13 mm can be diagnostic in cases of proven familial HCM
Echocardiographic recommendations	At initial evaluation, all patients should undergo transthoracic 2D and Doppler echocardiography, at rest and during Valsalva manoeuvre in the sitting and semi-supine positions and then on standing if no gradient is provoked to detect LVOTO. (1B) In symptomatic patients with HCM and a resting or provoked peak LV outflow tract gradient <50 mmHg, 2D and Doppler echocardiography during exercise in the standing, sitting (when possible), or semi-supine position are recommended to detect provocable LVOTO and exercise-induced mitral regurgitation. (1B)	In patients with suspected HCM, a transthoracic echocardiogram (TTE) is recommended in the initial evaluation. (1 B-NR) For patients with HCM and resting peak LVOT gradient <50 mmHg, a TTE with provocative manoeuvres is recommended. (1 B-NR) For symptomatic patients with HCM who do not have a resting or provocable outflow tract peak gradient ≥50 mmHg on TTE, exercise TTE is recommended for the detection and quantification of dynamic LVOTO. (1 B-NR)	TTE is recommended in patients with suspected HCM to establish the diagnosis, identify morphological subtype, evaluate haemodynamics, LV function and comorbidities, assess severity/distribution of LV hypertrophy, and detect LVOTO and MR. (I B) Stress echocardiography (Valsalva manoeuvre, standing) for detection of dynamic LVOTO in patients with peak LVOT gradient <50 mmHg at rest. (I C) Evaluate severity of heart failure, cardiac function and haemodynamics. (I C)
			Exercise TTE for detection of dynamic LVOTO in symptomatic patients with peak LVOT gradient <50 mmHg at rest. (IIa B)
CMR recommendations	Contrast-enhanced CMR is recommended in patients with HCM at initial evaluation. (1B) Contrast-enhanced CMR should be considered in patients with cardiomyopathy during follow-up to monitor disease progression and aid risk stratification and management. (IIaC)	For patients suspected of having HCM in whom echocardiography is inconclusive, CMR imaging is indicated for diagnostic clarification. (1 B-NR) For patients with LVH in whom there is a suspicion of alternative diagnoses, including infiltrative or storage disease as well as athlete's heart, CMR imaging is useful. (1 B-NR)	Cine CMR affords greater accuracy than echocardiography in identifying regions of LVH, mitral valve, or papillary muscle abnormalities. (I A) LGE pattern on CMR is useful to distinguish HCM from other LVH disease. (I B) Presence and extent of LGE is significantly associated with sudden cardiac death and all-cause death in HCM. (I A)
SCD risk assessment and prevention	Implantation of an ICD is recommended in patients who have survived a cardiac arrest due to VT or VF, or who have spontaneous sustained VT with haemodynamic compromise. (1B) The HCM Risk-SCD calculator is recommended as a method of estimating risk of sudden death at 5 years in patients aged ≥16 years for primary prevention. (1B) It is recommended that the 5-year risk of SCD be assessed at first evaluation and re-evaluated at 1–2 year intervals or whenever there is a change in clinical status. (1B) Implantation of an ICD should be considered in patients with an estimated 5-year risk of sudden death of ≥6%, following detailed clinical assessment that considers: (i) the lifelong risk of complications; (ii) competing mortality risk from the disease and comorbidities; (iii) the impact of an ICD on lifestyle, socio-economic status, and psychological health. (IIaB) In patients with LV apical aneurysms, decisions about primary prevention ICD should be based on an assessment of risk using the HCM Risk-SCD. (IIaB) Implantation of an ICD may be considered in individual patients with an estimated 5-year risk of SCD of between ≥4% and <6%, following detailed clinical assessment that takes into account the lifelong risk of complications and the impact of an ICD on lifestyle, socio-economic status, and psychological health. (IIbB)	A comprehensive, systematic non-invasive SCD risk assessment at initial evaluation and every 1–2 years thereafter is recommended and should include evaluation of these risk factors: (a) Personal history of cardiac arrest or sustained ventricular arrhythmias; (b) Personal history of syncope suspected by clinical history to be arrhythmic; (c) Family history in close relative of premature HCM-related sudden death, cardiac arrest, or sustained ventricular arrhythmias; (d) Maximal LV wall thickness, EF, LV apical aneurysm; (e) NSVT episodes on continuous ambulatory electrocardiographic monitoring. (1 B-NR) For patients with HCM who are not otherwise identified as high risk for SCD, or in whom a decision to proceed with ICD placement remains uncertain after clinical assessment, CMR imaging is beneficial to assess for maximum LV wall thickness, EF, LV apical aneurysm, and extent of myocardial fibrosis with LGE. (1 B-NR) For patients with HCM and previous documented cardiac arrest or sustained VT, ICD placement is recommended. (1 B-NR) For adult patients with HCM with ≥1 major risk factors for SCD, it is reasonable to offer an ICD. These are: (a) Sudden death judged definitively or likely attributable to HCM in ≥1 first-degree or close relatives who are ≤50 years of age; (b) Massive LVH ≥30 mm in any LV segment; (c) ≥1 recent episodes of syncope suspected by clinical history to be arrhythmic (i.e. unlikely to be of vasovagal aetiology, or related to LVOTO); (d) LV apical aneurysm with transmural scar or LGE; (e) LV systolic dysfunction (EF <50%). (2a B-NR)	Assessment of SCD risk at first evaluation and re-evaluation at 1–2 year intervals, or if change in clinical status. (I A) ICD implantation is indicated if the patient has a history of sustained VT or successful recovery from cardiac arrest due to VF or sustained VT. (I B) ICD implantation if ≥1 of following major risk factors: (a) recent cardiogenic or unexplained syncope; (b) maximum LV wall thickness ≥30 mm; (c) high risk patient judged by HCM Risk-SCD model as per ESC guideline. (IIa C) ICD implantation in presence of ≥2 major risk factors: (a) family history of SCD; (b) NSVT; (c) abnormal BP response during exercise. (IIa C) Consider ICD implantation in presence of ≥1 major risk factors and risk modifiers: Major risk factors: (a) family history of SCD; (b) NSVT; (c) abnormal BP response during exercise. Risk modifiers: (a) LVOTO, (b) extensive LGE on CMR, (c) dilated-phase HCM, (d) ventricular aneurysm. (IIa C) Consider ICD implantation in presence of a major risk factor OR risk modifiers: Major risk factors: ≥1 of family history of SCD, NSVT, abnormal BP response during exercise.
	For patients who are in the low-risk category (<4% estimated 5-year risk of SCD), the presence of extensive LGE (≥15%) on CMR may be considered in shared decision-making with patients about prophylactic ICD implantation, acknowledging the lack of robust data on the impact of scar quantification on the personalized risk estimates. (IIbB) For patients who are in the low-risk category (<4% estimated 5-year risk of SCD), the presence of LVEF <50% may be considered in shared decision-making with patients about prophylactic ICD implantation, acknowledging the lack of robust data on the impact of systolic dysfunction on the personalized risk estimates. (IIbB)	In select adult patients with HCM and without major SCD risk factors after clinical assessment, or in whom the decision to proceed with ICD placement remains otherwise uncertain, ICD may be considered in patients with extensive LGE by contrast-enhanced CMR imaging or NSVT present on ambulatory monitoring. (2b N-R) For patients who are ≥16 years of age with HCM, it is reasonable to obtain echocardiography-derived left atrial diameter and maximal LVOT gradient to aid in calculating an estimated 5-year sudden death risk that may be useful during shared decision-making for ICD placement. (2a B-NR)	Risk modifiers: LVOTO, extensive LGE on CMR, dilated-phase HCM, ventricular aneurysm. (IIb C) Beta blockers and/or amiodarone in patients with an ICD who have recurrent shocks. (I C)
Medical management of obstructive HCM	Non-vasodilating beta blockers, titrated to maximum tolerated dose, are recommended as first-line therapy to improve symptoms in patients with resting or provoked LVOTO. Verapamil or diltiazem are recommended in those unable to receive beta blockers. (I B) Disopyramide, titrated to maximum tolerated dose, is recommended in addition to a beta blocker (or, if not possible, with verapamil or diltiazem) to improve symptoms in patients with resting or provoked LVOTO. (I B) Cardiac myosin ATPase inhibitor (mavacamten), titrated to maximum tolerated dose with echocardiographic surveillance of LVEF, should be considered in addition to a beta blocker (or, if this is not possible, with verapamil or diltiazem) to improve symptoms in adult patients with resting or provoked LVOTO. (IIa A). It can be considered as monotherapy in individuals who are intolerant to beta blockers, calcium channel blockers and disopyramide. (IIa B)	In those with symptoms attributable to LVOTO non-vasodilating beta blockers are recommended. In those whom beta blockers are ineffective or not tolerated, substitution with verapamil or diltiazem is recommended. For patients with obstructive HCM who have persistent symptoms attributable to LVOTO despite beta blockers or non-dihydropyridine calcium channel blockers, adding a myosin inhibitor, or disopyramide (in combination with an atrioventricular nodal blocking agent), or SRT performed at experienced centres is recommended	Non-vasodilating beta blockers titrated to maximum tolerated dose, to improve symptoms in patients with resting or provoked LVOTO. Verapamil in those who are intolerant or contraindicated to beta blockers. (I B) Cibenzoline, or disopyramide, titrated to maximum tolerated dose, in addition to a beta blocker (or, if not possible, verapamil) to improve symptoms in patients with resting or provoked LVOTO. (I B) Beta blockers or verapamil in asymptomatic adults with resting or provoked LVOTO, to reduce LV pressure. (IIb C) Low-dose loop or thiazide diuretics with caution in symptomatic LVOTO to improve exertional dyspnoea. (IIb C) Diltiazem, titrated to maximum tolerated dose, in symptomatic patients with resting or provoked LVOTO, who are intolerant or have contraindications to beta blockers and verapamil, to improve symptoms. (IIb C)
Invasive management of obstructive HCM	It is recommended that SRT be performed by experienced operators working as part of a multidisciplinary team expert in the management of HCM. (IC) SRT to improve symptoms is recommended in patients with a resting or maximum provoked LVOT gradient of ≥50 mmHg who are in NYHA/Ross functional class III–IV, despite maximum tolerated medical therapy (IB) or those with exertional syncope. (IIaC) Septal myectomy, rather than ASA, is recommended in those with other lesions requiring surgical intervention (e.g. mitral valve abnormalities). (IC) Mitral valve repair or replacement should be considered in symptomatic patients with a resting or maximum provoked LVOTO gradient ≥50 mmHg and moderate-to-severe mitral regurgitation that cannot be corrected by SRT alone. (IIaC)	In patients with obstructive HCM who remain symptomatic despite GDMT, SRT in eligible patients, performed at experienced HCM centres, is recommended for relieving LVOTO. (1 B-NR) In symptomatic patients with obstructive HCM who have associated cardiac disease requiring surgical treatment surgical myectomy, performed at experienced HCM centres, is recommended (1 B-NR) In patients with obstructive HCM who remain severely symptomatic, despite GDMT and in whom surgery is contraindicated or the risk is considered unacceptable because of serious comorbidities or advanced age, alcohol septal ablation in eligible patients, performed at experienced HCM centres, is recommended (1 C-LD)	SRT to be performed by experienced operators working as part of an MDT expert in the management of HCM. (I C) SRT to improve symptoms in patients with a resting or maximum provoked LVOT ≥50 mmHg, who are NYHA III-IV, despite maximum tolerated medical therapy. (I B) SRT in patients with chest pain or recurrent exertional syncope caused by a resting or maximum provoked LVOTO ≥50 mmHg despite optimal medical therapy. (I B) Septal myectomy, rather than PTSMA, when SRT is indicated, and in patients with other lesions requiring surgical intervention (e.g. mitral valve abnormalities). (IIa C) PTSMA in eligible patients with drug-refractory symptoms when surgery in contraindicated or risk is not acceptable. (IIa C) Mitral valve repair or replacement in patients with a resting or maximum provoked LVOTO gradient ≥50 mmHg and moderate-severe MR not caused by SAM of the mitral valve alone. (IIa C)
Management of non-obstructive HCM	Beta blockers and calcium antagonists (verapamil or diltiazem) should be considered to improve symptoms in patients with angina-like chest pain even in the absence of LVOTO or obstructive CAD. (IIaC) Oral nitrates or ranolazine may be considered to improve symptoms in patients with angina-like chest pain, even in the absence of obstructive CAD, if there is no LVOTO. (IIaC)	In patients with non-obstructive HCM with preserved EF and symptoms of exertional angina or dyspnoea, beta blockers or non-dihydropyridine calcium channel blockers are recommended. (1 C-LD) In patients with non-obstructive HCM with preserved EF, it is reasonable to add oral diuretics when exertional dyspnoea persists despite the use of beta blockers or non-dihydropyridine calcium channel blockers. (2A C-EO)	Use of beta blockers and verapamil in patients with NYHA class I symptoms (in HFpEF) to improve diastolic function. (IIb C) Use of beta blockers and verapamil or diltiazem in patients with NYHA class II-IV symptoms to improve symptoms. (I B) Use of low-dose diuretics (loop or thiazide) in patients with NYHA class II-IV symptoms (in HFpEF) to improve congestive symptoms. (I C) ACEi or ARB and beta blockers in patients with dilated-phase HCM (in HFmrEF and HFrEF). (I C) In patients with dilated-phase HCM and NYHA class II-IV symptoms, consider addition of an MRA and diuretic. (IIa C)
Management of atrial fibrillation in HCM	Oral anticoagulation in order to reduce the risk of stroke and thrombo-embolic events is recommended in all patients with HCM	In patients with HCM and clinical AF, anticoagulation is recommended with direct-acting oral anticoagulants (DOACs) as first-line option and vitamin K antagonists as second-line option, independent of CHA2DS2-VASc score. (1 B-NR) In patients with HCM and subclinical AF detected by internal or external cardiac device or monitor of >24 h duration for a given episode, anticoagulation is recommended with DOACs as first-line option and vitamin K antagonists as second-line option, independent of CHA2DS2-VASc score. (1 C-LD) In patients with HCM and subclinical AF detected by internal or external device or monitor, of >5 min duration but <24 h duration for a given episode, anticoagulation with DOACs as first-line option and vitamin K antagonists as second-line option can be beneficial, taking into consideration duration of AF episodes, total AF burden, underlying risk factors, and bleeding risk. (2a C-LD)	Oral anticoagulation to prevent thromboembolism. (I B)
Genetic testing and family screening	Genetic testing is recommended in patients fulfilling diagnostic criteria for cardiomyopathy in cases where it enables diagnosis, prognostication, therapeutic stratification, or reproductive management of the patient, or where it enables cascade genetic evaluation of their relatives who would otherwise be enrolled into long-term surveillance. (IB) Genetic testing in patients with a borderline phenotype not fulfilling diagnostic criteria for a cardiomyopathy may be considered only after detailed assessment by specialist teams. (IIbC) It is recommended that cascade genetic testing, with pre- and post-test counselling, is offered to adult at-risk relatives if a confident genetic diagnosis (i.e. a P/LP variant) has been established in an individual with cardiomyopathy in the family (starting with first-degree relatives if available, and cascading out sequentially). (IB)	In patients with HCM, genetic testing is beneficial to elucidate the genetic basis to facilitate the identification of family members at risk for developing HCM (cascade testing). (1 B-NR) In patients with an atypical clinical presentation of HCM or when another genetic condition is suspected to be the cause, a workup including genetic testing for HCM and other genetic causes of unexplained cardiac hypertrophy (‘HCM phenocopies’) is recommended. (1 B-NR) In first-degree relatives of patients with HCM, both clinical screening (ECG and 2D echocardiogram) and cascade genetic testing (when a pathogenic/likely pathogenic variant has been identified in the proband) should be offered. (1 B-NR)	Genetic testing to confirm the diagnosis in the presence of symptoms and findings of disease suggestive of secondary cardiomyopathy. (I B) Genetic testing in HCM patients when it enables cascade genetic screening of their family members. (IIa B) Genetic testing in HCM patients if family screening is difficult to perform. (IIb B) Genetic testing for the assessment of SCD risk in HCM patients. (IIb B) Creating a family tree for patients with HCM, with clinical evaluation of first-degree relatives and long-term follow up in relatives who have the same definite disease-causing mutation as the proband. (I C) Cascade genetic screening in first-degree relatives of patients with a definite disease-causing mutation. (IIa B)
	Diagnostic genetic testing is not recommended in a phenotype-negative relative of a patient with cardiomyopathy in the absence of a confident genetic diagnosis (i.e. a P/LP variant) in the family. (IIIC)	For patients with HCM who have undergone genetic testing and were found to have no pathogenic variants (i.e. harbour only benign or likely benign variants), cascade genetic testing of the family is not useful. (3 B-NR)	Discontinuance of ongoing clinical follow-up in genotype-negative relatives of patients with a definite disease-causing mutation. (IIa B) Genetic testing is not recommended in relatives when the HCM proband does not have a definitive disease-causing mutation. (III C)
Exercise recommendations in HCM	Regular low- to moderate-intensity exercise is recommended in all able individuals with HCM. (I C) High-intensity exercise and competitive sport should be considered in genotype-positive/phenotype-negative individuals who seek to do so. (IIaC) High-intensity exercise and competitive sport may be considered in asymptomatic low-risk individuals with morphologically mild hypertrophic cardiomyopathy in the absence of resting or inducible left ventricular outflow obstruction and exercise-induced complex ventricular arrhythmias. (IIbB) High-intensity exercise, including competitive sport, is not recommended in high-risk individuals and in individuals with left ventricular outflow tract obstruction and exercise-induced complex ventricular arrhythmias. (IIIC)	For patients with HCM, mild- to moderate-intensity recreational exercise is beneficial for overall health in keeping with physical activity guidelines for the general population. (1 B-R) In individuals who are genotype-positive, phenotype-negative for HCM, participation in competitive sports of any intensity is reasonable. (2a B-NR) For most patients with HCM, universal restriction from vigorous physical activity or competitive sports is not indicated. (3 B-NR) In patients with HCM, ICD placement for the sole purpose of participation in competitive sports should not be performed. (3 C-EO)	Avoidance of competitive sports to prevent SCD. (I C) Consider exercise therapy to improve exercise capacity. (IIb C) In patients with advanced deconditioning and reduced physical functioning, consider resistance training to improve ADLs and QOL. (IIb C)
Reproductive and pregnancy management in HCM	Counselling on the risk of disease inheritance is recommended for all men and women before conception. (I C)	In affected families with HCM, pre-conceptional and prenatal reproductive and genetic counselling should be offered. (1 B-NR)	No formal recommendations
	Vaginal delivery is recommended in most women with cardiomyopathies, unless there are obstetric indications for caesarean section, severe heart failure (EF <30% or NYHA class III–IV), or severe outflow tract obstructions, or in women presenting in labour on oral anticoagulants. (I C) It is recommended that medication be carefully reviewed for safety in advance of pregnancy and adjusted according to tolerability in pregnancy. (I C) Therapeutic anticoagulation with LMWH or VKAs according to the stage of pregnancy is recommended for patients with AF. (I C) Continuation of beta blockers should be considered during pregnancy in women with cardiomyopathies, with close follow-up of foetal growth and of the condition of the neonate, and if benefits outweigh risks. (IIa C)	In most pregnant women with HCM, vaginal delivery is recommended as the first-choice delivery option. (1 C-LD) For pregnant women with HCM and AF or other indications for anticoagulation, low-molecular-weight heparin or vitamin K antagonists (at maximum therapeutic dose of <5 mg daily) are recommended for stroke prevention. (1 B-NR) In pregnant women with HCM, selected beta blockers should be administered for symptoms related to outflow tract obstruction or arrhythmias, with monitoring of foetal growth. (1 C-LD) In pregnant women, use of mavacamten is contraindicated due to potential teratogenic effects. (3 C-EO)	

Recommendations:

ESC and JSC/JHFS:

*Level of evidence*:

A = data derived from multiple randomized clinical trials or meta-analysis. B = data derived from a single randomized trial or non-randomized studies. C = only consensus opinion of experts, and/or small studies, retrospective studies, registries.

*Class of recommendation*:

I, evidence and/or general agreement that the procedure or treatment is beneficial, useful, and effective; Class II: conflicting evidence and/or a divergence of opinion about the usefulness/efficacy of a procedure or treatment; IIa, weight of evidence/opinion is in favour of usefulness/efficacy; IIb, usefulness/efficacy is less well established by evidence/opinion; III, evidence and/or general agreement that the procedure/treatment is not useful/effective and in some cases may be harmful.

AHA/ACC/AMSSM/HRS/PACES/SCMR:

*Level of evidence*:

A = (high quality evidence). B = moderate quality evidence—B-R = randomized study, B-NR = non-randomized. C = C-LD = limited data, C-EO = consensus of expert opinion.

*Class of recommendation*:

Class I = benefit >>> risk; Class IIa = benefit >> risk, Class IIb = benefit ≥ risk; class III = risk ≥ benefit.

ACEi, angiotensin-converting enzyme inhibitor; ADLs, activities of daily living; AF, atrial fibrillation; ARB, angiotensin II receptor blocker; ASA, alcohol septal ablation; BP, blood pressure; CAD, coronary artery disease; CMR, cardiac magnetic resonance; DOACs, direct-acting oral anticoagulants; ECG, electrocardiogram; EF, ejection fraction; GDMT, guideline-directed medical therapy; HCM, hypertrophic cardiomyopathy; HF, heart failure; HFmrEF, heart failure with mid-range ejection fraction; HFrEF, heart failure with reduced ejection fraction; HFpEF, heart failure with preserved ejection fraction; ICD, implantable cardioverter-defibrillator; LGE, late gadolinium enhancement; LMWH, low molecular weight heparin; LV, left ventricle/ventricular; LVH, left ventricular hypertrophy; LVEF, left ventricular ejection fraction; LVOTO, left ventricular outflow tract obstruction; MR, mitral regurgitation; MRA, mineralocorticoid receptor antagonist; NSVT, non-sustained ventricular tachycardia; NYHA, New York Heart Association; P/LP variant, pathogenic/likely pathogenic variant; PTSMA, percutaneous transluminal septal myocardial ablation; QOL, quality of life; RV, right ventricle; SAM, systolic anterior motion; SCD, sudden cardiac death; SRT, septal reduction therapy; TTE, transthoracic echocardiogram; VF, ventricular fibrillation; VKAs, vitamin K antagonists; VT, ventricular tachycardia.

a Relationship with industry is reported by any group member.

b A group member is recused when a relevant area is under discussion.

### Areas of agreement

#### HCM definition and diagnostic criteria

All guidelines agree that HCM is defined by the presence of increased LV wall thickness or hypertrophy and that this cannot be explained by abnormal loading conditions or any other cardiac, metabolic, storage, infiltrative, or systemic disease. In their definitions, the AHA/ACC/AMSSM/HRS/PACES/SCMR and JCS/JHFS guidelines also reference that a pathogenic mutation should be identified.

All guidelines use a ≥15 mm end-diastolic wall thickness in any segment of the LV as the diagnostic threshold for HCM in adults. All guidelines suggest a lower threshold (≥13 mm) in individuals who have a family member with a confirmed diagnosis of HCM. The ESC guidelines comment that lesser degrees of wall thickening (13–14 mm) require evaluation of other features including family history, genetic findings, and ECG abnormalities. The AHA/ACC/AMSSM/HRS/PACES/SCMR guidelines state that this more limited degree of hypertrophy (13–14 mm) can be diagnostic in cases where a pathogenic/likely pathogenic (P/LP) sarcomeric gene variant is identified.

#### Echocardiographic recommendations for HCM

All guidelines agree that two-dimensional transthoracic echocardiography should be performed in the initial evaluation of an individual with suspected HCM. There is also consensus that provocative manoeuvres (e.g. Valsalva) should be performed to detect peak left ventricular outflow tract (LVOT) gradient and characterize LVOT obstruction. For HCM patients who are symptomatic and have a resting LVOT gradient of ≤50 mmHg, all guidelines recommend performing an echocardiogram during exercise in order to detect and quantify dynamic LVOT obstruction.

#### Medical management of HCM

All guidelines advocate the use of non-dilating beta blockers, or if these are not tolerated, non-dihydropyridine calcium channel blockers, in individuals with HCM who are symptomatic due to LVOT obstruction. In patients with non-obstructive HCM and symptoms of angina-like chest pain or dyspnoea, all guidelines recommend beta blockers or non-dihydropyridine calcium channel blockers.

Regarding the management of atrial fibrillation, all guidelines agree that lifelong, oral anticoagulation is recommended for all patients with HCM, irrespective of CHA2DS2-VASc scoring. The AHA/ACC/AMSSM/HRS/PACES/SCMR guidelines specify that direct oral anticoagulants are first line over vitamin K antagonists (e.g. warfarin).

#### Secondary prevention implantable cardioverter-defibrillator therapy in HCM

All guidelines agree that implantation of a secondary prevention implantable cardioverter-defibrillator (ICD) is recommended in individuals with HCM who have suffered a cardiac arrest due to ventricular arrhythmia or have a documented history of sustained ventricular tachycardia (VT).

#### Septal reduction therapy in HCM

All guidelines recommend that septal reduction therapy (SRT) is performed by experienced operators in experienced centres within the framework of multidisciplinary teamwork. The ESC and JCS/JHFS guidelines recommend SRT to be performed in individuals with an LVOT gradient (resting or provoked) ≥50 mmHg who are in New York Heart Association (NYHA) Class III–IV despite maximal medical therapy. The AHA/ACC/AMSSM/HRS/PACES/SCMR guidelines recommend that SRT should be performed in eligible patients with obstructive HCM who remain symptomatic despite goal-directed medical therapy. The guideline's definition of ‘eligible’ is as follows: (i) clinical: severe dyspnoea or chest pain (usually NYHA class III or IV), or occasionally other exertional symptoms (e.g. syncope, near syncope), when attributable to LVOT obstruction, that interferes with everyday activity or quality of life despite optimal medical therapy; (ii) haemodynamic: dynamic LVOT gradient at rest or with physiologic provocation with approximate peak gradient of ≥50 mmHg, associated with septal hypertrophy and systolic anterior motion of the mitral valve; and (iii) anatomic: targeted anterior septal thickness is sufficient to perform the procedure.

Regarding the choice of SRT, septal myectomy or alcohol septal ablation (ASA), all guidelines agree that septal myectomy rather than ASA should be performed in individuals with other lesions requiring surgical intervention. In those where SRT is indicated but surgery is contraindicated or associated with unacceptable risk, then ASA is recommended.

#### Genetic testing and family screening in HCM

The approach to genetic testing in both the proband (index HCM case) and family screening thereafter is consistent across guidelines. It is agreed that genetic testing for a patient with HCM is beneficial to elucidate its genetic basis to facilitate cascade genetic screening of family members if required. All guidelines also recommend that genetic testing is beneficial in patients with a borderline phenotype or atypical presentation of HCM or when another genetic condition may be the cause of unexplained cardiac hypertrophy (‘HCM phenocopies’). Diagnostic genetic testing in phenotype-negative family members is not recommended if a definitive disease-causing mutation (i.e. P/LP variant) is not identified in the HCM proband.

#### Reproductive and pregnancy considerations in HCM

The ESC and AHA/ACC/AMSSM/HRS/PACES/SCMR guidelines are concordant in their recommendations, namely that reproductive and genetic counselling should be offered prior to conception; vaginal delivery is recommended as the mode of delivery in most women with HCM, medication should be reviewed for its suitability in advance of pregnancy with the AHA/ACC/AMSSM/HRS/PACES/SCMR guideline explicitly noting that the use of mavacamten is contraindicated due to teratogenicity, and that therapeutic anticoagulation should be with low-molecular-weight heparin or vitamin K antagonists. The JCS/JHFS guidelines do not make any formal recommendations on this aspect of management in individuals with HCM.

#### Exercise recommendations in HCM

The recommendations regarding exercise and physical activity are sparser in the JCS/JHFS guidelines compared to the ESC and AHA/ACC/AMSSM/HRS/PACES/SCMR guidelines. Both latter guidelines have near equivalence in defining light intensity, moderate intensity, and high-intensity/vigorous exercise based on maximum heart rate achieved. Both guidelines recommend that mild-to-moderate level intensity exercise is beneficial in all able individuals with HCM. Similarly, they advise that genotype-positive/phenotype-negative individuals are not excluded from high-intensity exercise and competitive sport should they seek to do so.

However, differences were observed in the recommendations for high-intensity exercise.

The ESC guidelines are explicit in stating that high-intensity exercise, including competitive sport, is not recommended in high-risk individuals and in individuals with LV outflow tract obstruction and exercise-induced complex ventricular arrhythmias. The AHA/ACC/AMSSM/HRS/PACES/SCMR guidelines state that for most patients with HCM, universal restriction from vigorous physical activity or competitive sport is not indicated, although a comprehensive evaluation and shared-decision making process with an expert professional is recommended prior to participation. The AHA/ACC/AMSSM/HRS/PACES/SCMR guidelines also advocate that ICD placement for the sole purpose of participation in competitive sports should not be performed in patients with HCM, something which is not mentioned in any of the other guidelines.

### Areas of disagreement

#### Cardiovascular magnetic resonance imaging recommendations for HCM

There is variability across all three guidelines’ recommendations for the use of cardiovascular magnetic resonance (CMR) imaging in HCM. The ESC guidelines state that CMR with late gadolinium enhancement (LGE) imaging is recommended in patients with HCM at their initial evaluation and that it is superior to echocardiography in the detection of LV apical and anterolateral hypertrophy, aneurysms, and thrombi. It further suggests that CMR should be considered during follow-up to monitor progression and aid risk stratification. The AHA/ACC/AMSSM/HRS/PACES/SCMR guidelines recommend that for patients suspected of having HCM in whom echocardiography is inconclusive, CMR imaging is indicated for diagnostic clarification, and it is useful in patients with LV hypertrophy in whom there is a suspicion of an alternative diagnosis (e.g. athlete's heart, storage diseases, infiltrative diseases). In the AHA/ACC/AMSSM/HRS/PACES/SCMR guidelines, CMR imaging is further referenced in the context of sudden cardiac death (SCD) risk assessment and prevention as being beneficial in patients not otherwise identified as high risk for SCD, or in whom a decision to proceed with ICD placement remains uncertain after clinical assessment, in order to assess maximum LV wall thickness, ejection fraction, LV apical aneurysm, and LGE extent.

The JCS/JHFS guidelines provide recommendations regarding the utility of CMR in evaluating HCM rather than, as per the other guidelines, when it should be used in the diagnostic pathway. It states that CMR affords greater accuracy in identifying regions of LV hypertrophy compared to echocardiography; that the pattern of LGE on CMR is useful in distinguishing HCM from other hypertrophic diseases; and that the presence and extent of LGE is significantly associated with SCD and all-cause death in HCM.

#### Sudden cardiac death risk assessment and primary prevention ICD therapy in HCM

Risk prediction for primary prevention ICD is based on the systematic assessment of risk factors in all patients. The ESC guideline advocates for the use of the HCM Risk-SCD calculator, which estimates the risk of sudden death within 5 years for individuals with HCM. The HCM-Risk SCD calculator considers the following parameters: age, maximum wall thickness, left atrial diameter, maximum LVOT gradient, family history of SCD, recorded non-sustained VT, and unexplained syncope. It is recommended that the risk score be used at initial assessment and re-evaluated at 1–2-year intervals or whenever a change in clinical status occurs. Recommendations on subsequent decisions regarding implantation of an ICD are made, in part, based on the risk calculator output. The ESC guidelines recommend that implantation of an ICD should be considered if the 5-year risk of SCD is ≥6% and that it may be considered on an individual basis in those with a risk score between 4% and 6%. ICD implantation may also be considered in individuals with a risk score of <4% but extensive LGE (≥15%) on CMR or LVEF <50%.

The AHA/ACC/AMSSM/HRS/PACES/SCMR guidelines, whilst also advocating assessment of SCD risk at initial evaluation and every 1 or 2 years thereafter, recommend a systematic assessment, including evaluation of the following risk factors: a personal history of syncope suspected by clinical history to be arrhythmic, a family history of premature HCM-related sudden death/cardiac arrest/sustained ventricular arrhythmias; maximal wall thickness, ejection fraction <50%, and presence of an apical aneurysm; non-sustained VT on continuous ambulatory electrocardiographic monitoring, with it carrying greater weight as a risk maker with longer, faster, and more frequent documented episodes. This contrasts with the current ESC guidance which does not consider the burden of non-sustained VT as influencing SCD risk. For decision-making around ICD implantation, the AHA/ACC/AMSSM/HRS/PACES/SCMR guidelines provide a linked set of more specific criteria which constitute ‘major’ risk factors for SCD, the presence of ≥1 indicating that ICD implantation should be offered. These are: sudden death judged definitively or likely attributable to HCM in ≥1 first-degree or close relatives who are ≤50 years of age; LV hypertrophy ≥30 mm in any LV segment; ≥1 recent episodes of syncope suspected by clinical history to be arrhythmic (i.e. unlikely to be of vasovagal aetiology, or related to LVOT obstruction); LV apical aneurysm with transmural scar or LGE; LV systolic dysfunction (EF <50%). The ESC guidelines do not consider the presence of LV apical aneurysm alone to be a sufficiently strong predictor of SCD to guide primary prevention ICD implantation decisions.

The JCS/JHFS guideline recommendations on ICD implantation are as follows: ICD implantation is recommended if ≥1 of the following major risk factors are present: recent cardiogenic syncope; maximum LV wall thickness ≥30 mm; an HCM Risk-SCD score of ≥6%. ICD implantation is also recommended if ≥2 of the following major risk factors are present: family history of SCD; non-sustained VT; abnormal blood pressure response during exercise. If any one of these risk factors is present in combination with LVOT obstruction, extensive LGE on CMR, dilated-phase HCM or ventricular aneurysm, then an ICD should be considered.

Regarding the choice of defibrillator device, both the ESC guidelines and the AHA/ACC/AMSSM/HRS/PACES/SCMR suggest that subcutaneous defibrillators should be considered as an alternative to a transvenous device in individuals when pacing therapy for bradycardia, cardiac resynchronization, or anti-tachycardia pacing is not anticipated. The JCS/JHFS guidance states that subcutaneous ICDs may be more beneficial in younger individuals, but do not make any recommendations beyond this.

#### Medical management of HCM

The ESC and AHA/ACC/AMSSM/HRS/PACES/SCMR guidelines recommend the addition of disopyramide or a cardiac myosin ATPase inhibitor (e.g. mavacamten) in individuals whose symptoms of LVOT obstruction are incompletely controlled despite beta blockers or non-dihydropyridine calcium channel blockers. It should be noted that this comes as a Class IIa recommendation in the ESC guidelines and as a Class 1 indication in the AHA/ACC/AMSSM/HRS/PACES/SCMR guidelines. By contrast, the JCS/JHFS guideline, due to its release date, does not reference the use of myosin inhibitors. It recommends cibenzoline as well as disopyramide—both are class 1a antiarrhythmic drugs.

For patients with non-obstructive HCM experiencing angina-like chest pain, the ESC guidelines recommend consideration of oral nitrates or ranolazine as additional agents to beta blockers or calcium channel blockers, even in the absence of coronary artery disease. The AHA/ACC/AMSSM/HRS/PACES/SCMR and JCS/JHFS guidelines strongly recommend the use of diuretics to individuals with exertional dyspnoea and non-obstructive HCM and recommend cautious consideration in obstructive HCM.

## Discussion

**Figure 2:**
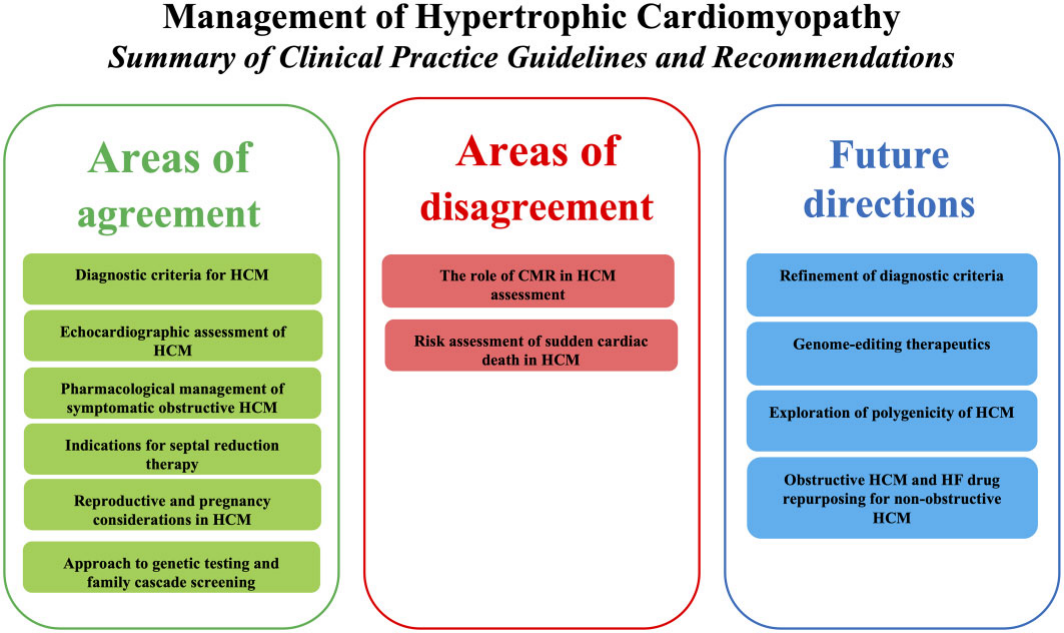
Overview of areas of agreement, disagreement, and future directions in the diagnosis and management of HCM.

Following a systematic search, three guidelines on the diagnosis and management of HCM were identified, all of which were robustly developed. These guidelines were authored by European (ESC; 2023), American (AHA/ACC/AMSSM/HRS/PACES/SCMR; 2024), and Japanese (JCS/JHFS; 2018) cardiovascular societies. There was broad consensus on echocardiographic recommendations, the medical and invasive management of HCM, the application of genetic testing and family screening, and exercise and reproductive recommendations in HCM. There were areas of variability in the definition and diagnostic criteria for HCM, CMR recommendations and assessment of SCD risk and primary prevention strategies. Due to the JCS/JHFS being an older guideline, there are no recommendations on the use of cardiac myosin ATPase inhibitors. A summary of points of agreement, disagreement, and current evidence gaps/areas for future research is provided in *Figure 2*.

### Diagnostic criteria

All guidelines are relatively similar regarding the diagnostic criteria for HCM, which centres on an end-diastolic LV maximal wall thickness of ≥15 mm in adults. It is notable that this is a standalone threshold, without adjustment for ethnicity, sex, or body size. A paper by Le and colleagues in a Singaporean cohort, having noted that east Asian individuals have smaller heart sizes than white individuals, demonstrated that reducing wall thickness thresholds to 10 mm in males and 12 mm in females did not affect HCM diagnosis specificity (100%) but significantly improved sensitivity (86%).^9^ Similar observations have been made in women, showing a 1–2 mm lower absolute wall thickness when compared to men.^10^ Moreover, in cases of apical HCM, to attain a wall thickness of 15 mm there needs to be, in relative terms, substantially more hypertrophy compared to the base due to natural apical tapering of the myocardium. Hughes *et al.* have suggested novel thresholds for apical HCM diagnosis based on segment-based, body surface area-indexed apical wall thickness.^11^ The ESC guideline notes that ‘lesser degrees of wall thickening (13-14 mm) requires evaluation of other features’ which may tacitly acknowledge the limitations of singular, ‘one-size-fits-all’ wall thickness threshold.

### The use of CMR imaging

There is some variation between the guidelines regarding recommendations for CMR imaging. Whilst the ESC guideline recommends that CMR imaging is employed at initial evaluation and should be considered as part of routine follow-up for progression monitoring, and the JCS/JHFS guideline states that CMR affords greater accuracy than echocardiography for identifying hypertrophy and other abnormalities, the AHA/ACC/AMSSM/HRS/PACES/SCMR guideline reserves recommending CMR for when echocardiography is inconclusive or where there is a suspicion of an alternative diagnosis. This is in keeping with a generally observed trend that CMR is less represented across clinical guidelines produced by the AHA and ACC when compared with guidelines from the ESC.^12^ Per Organisation for Economic Co-operation and Development (OECD) data, the United States is second only to Japan with respect to the number of magnetic resonance imaging (MRI) scanners it has per million inhabitants (40.4 scanners per million in the USA vs. 57.3 scanners per million in Japan), however CMR examinations account for only 1% of scans performed.^13,14^

### SCD risk assessment

Determining the risk of sudden death and decision-making regarding ICD implantation is a critical component in the ongoing management and follow-up of individuals with HCM. Whilst there is variation in approach to SCD risk assessment between the guidelines, it should be emphasized that these are primarily differences of nuance with the risk factors which underpin these approaches being broadly the same and being defined in similar ways. Here, we examine two areas which encapsulate this variation: first, how ongoing SCD risk is assessed and second, the incorporation of LGE imaging data into the risk assessment process.^15^

The ESC guideline, as in its previous 2014 iteration,^16^ utilizes the HCM Risk-SCD risk prediction model to provide a quantitative risk estimate of 5-year risk of SCD based on a number of phenotypic inputs. It has been validated in independent cohorts where it has been demonstrated that the predicted estimates are concordant with the observed SCD risk in patients deemed either high- or low-risk. The ESC advocates that utilization of the prediction model is the first step in assessing SCD risk.

This contrasts with the AHA/ACC/AMSSM/HRS/PACES/SCMR and JCS/JHFS guidelines. Whilst some studies, referenced above, have validated the HCM Risk-SCD score, there have been other studies which are more critical. A study by Maron *et al.* on the HCM Risk-SCD score noted that the occurrence rate for events was high for patients designated to be at high risk; with more than half of those with a sudden death event were deemed to be low risk.^17^ This study retrospectively examined 1629 patients from two tertiary referral centres based in the United States. It is important to note that this study did not restrict itself to a follow-up period of 5 years following initial risk assessment, which is the timeframe for which the score is validated. As such, the findings of Maron *et al.* may be explained by unaccounted changes in risk profile beyond 5 years from baseline evaluation.^18^ A further study involving 206 Japanese patients showed that although, again, the calculator was useful for predicting events in the high-risk group, a considerable number of sudden deaths occurred in patients judged to be in the low or intermediate risk group.^19^ The JCS/JHFS guideline takes an approach whereby SCD risk and thereby decisions for ICD implantation are taken on the basis of a set of clinical criteria and eschews the use of a risk calculator as a first step. Within the JCS/JHFS guidelines, it is notable that abnormal blood pressure response to exercise remains a major SCD risk factor whilst it has been removed as a routine component of SCD risk assessment in the European and American guidelines. Whilst an abnormal blood pressure response (a lack of rise in blood pressure during exercise of at least 20 mmHg) has been shown to have a potent association with SCD in high-risk adult patients ≤40 years of age, it has low positive predictive accuracy and an unknown prognostic significance in those >40 years.^6,20^

The approach of assessing risk factors rather than utilizing a risk score calculator is also followed in the AHA/ACC/AMSSM/HRS/PACES/SCMR guidelines. The writing committee explains that, given that individual patients may consider risk estimates for SCD differently, management recommendations should not be assigned to pre-specified thresholds as the arbiter by which to guide ICD implantation measures. They also remark that ‘contemporary’ markers of SCD risk in HCM do not form part of the modelling—parameters such as LV apical aneurysm, presence of LGE and LV systolic dysfunction. Here, it might be noted that many of these markers are either better or solely assessed using CMR; perhaps an implicit acknowledgement of its utility in HCM assessment despite the explicit recommendations. Whilst the AHA/ACC/AMSSM/HRS/PACES/SCMR guidelines consider the presence of LV apical aneurysm as an independent SCD risk factor for guiding primary prevention decisions, the ESC guideline recommends that these decisions should not solely be based on the presence of an LV apical aneurysm—this is one of the more overt points of difference between the two contemporary guidelines. The ESC guidelines point to the fact that the two studies from which evidence around apical aneurysm and SCD risk arises from are both retrospective, from a small number of centres (one a single-centre study, the other a two-centre study) and a high prevalence of confounders in individuals with events such as previous ventricular arrhythmia and impaired LV systolic function.^21,22^

The presence of myocardial fibrosis as detected by LGE imaging is incorporated into recommendations differently by the guidelines. The JCS/JHFS and AHA/ACC/AMSSM/HRS/PACES/SCMR guidelines advocate for its use in the decision-making process regarding ICD implantation. However, the ESC guideline recommends that for individuals categorized as low risk by the HCM Risk-SCD score (≤4%), the presence of ≥15% LGE may be considered in the decision-making about ICD implantation, ‘acknowledging the lack of robust data on the impact of scar quantification on personalized risk estimates’.

To date, there are several studies that appear to suggest that the presence of LGE is significant in determining onward risk of SCD in HCM patients. Chan *et al.* demonstrated that HCM patients with LGE of ≥15% of LV mass demonstrated a two-fold increase in SCD event risk in those otherwise considered to be at low risk.^23^ There are three aspects of the work of Chan *et al.* that are particularly noteworthy, however: first, the study's assertion around the predictive power for LGE of SCD is driven by including ICD discharges as equivalent to SCD events, which has been demonstrated to potentially overestimate sudden death rates; second, the majority of the event cohort were likely not truly low-risk as the majority had at least one conventional risk factors for SCD; and third, when considering the 20 individuals that had SCD or cardiac arrest, 80% of these had trivial (<5%) LGE on CMR. It has further been pointed out that risk assessment was incomplete in a significant proportion of this cohort in whom ambulatory ECG monitoring and other data were missing.^24^ Meta-analyses by Weng (2993 patients) and Briasoulis (3067 patients) investigating the predictive value of LGE on CMR for SCD in HCM have demonstrated that quantitative LGE exhibits prognostic value in SCD prediction independent of baseline characteristics both in all HCM patients and those assigned as low-risk.^25,26^ The work of Chan *et al.* forms the largest contributing cohort in both of these meta-analyses; its limitations may have important consequences for the validity of these results. The ESC guideline writing group explicitly acknowledges the discrepant approach surrounding myocardial fibrosis and risk assessment, suggesting that the population groups examined in the relevant studies may not be representative of the overall broad spectrum of HCM. Specifically, it points to the HCMR study, a prospective CMR study of 2755 patients, recorded only 24 deaths from any cause at a median follow-up of 36 months.^27^ It also references debate surrounding the method of LGE quantification as a limitation noting that only the two-standard deviation technique has been validated histologically.^28^ However, a recent meta-analysis comparing quantitative LGE methods in HCM (5550 patients) demonstrated that the different quantification techniques all have comparable accuracy in predicting SCD.^29^

### Evidence gaps and future directions in HCM

In addition to the above areas, there are other emerging fields in the study of HCM which may influence recommendations to be incorporated in future iterations of guidelines. First, due to the timing of guideline publication, the ESC and AHA/ACC/AMSSM/HRS/PACES/SCMR only reference mavacamten in the context of myosin inhibitors. Since their publication, phase 3 data for second-in-class molecule aficamten (shorter half-life compared to mavacamten) have been published,^30^ as well as long-term (3.5 years) follow-up and ‘real-world’ experience with mavacamten^31,32^; the burgeoning research output around utility of myosin inhibitors will form a key component when synthesizing future guidelines. Second, new therapies beyond myosin inhibitors – particularly in the realm of genome editing with CRISPR/Cas9 technology – are emerging as a prominent area of research in recent years. Two groups have recently published on *in vivo* correction of the HCM-linked c.1208G > A (p.R403Q) variant in β-myosin (*MYH7*) using base editing technology. Utilizing humanized mouse models exhibiting HCM traits, these groups have demonstrated the ability to correct the target mutation in cardiomyocytes and reduce HCM characteristics with normalization of echocardiographic parameters.^33,34^ In the gene replacement sphere, work has moved beyond the mouse model. The MyPEAK-1 phase 1b/2 clinical trial is ongoing in adults with MYBPC3-associated HCM which aims to deliver a functional MYBPC3 gene to myocardial cells via an adeno-associated virus-based gene therapy. Similar gene replacement trials are ongoing in HCM phenocopy conditions such as the Danon disease and Friedreich's ataxia cardiomyopathy.^35,36^ The interest in this potentially curative approach has further been highlighted in the award of £30 million by the British Heart Foundation to the CureHeart multinational research partnership whose stated aim is to develop transformative and potentially curative gene therapy approaches for inherited cardiomyopathies.^37^ Third, advances in our understanding of the genetic architecture of HCM. Given that a genetic aetiology is undetermined in approximately 50% of HCM cases, it is increasingly recognized that polygenicity is an important component in HCM.^38^ Recent studies have suggested that common genetic variation may account for up to 34% of heritability in HCM and the utility of common variants in prediction via polygenic risk scores is certain to be deeply explored going forward.^39^ Finally, tackling symptom management in individuals with non-obstructive HCM which remains clinically challenging. There are ongoing clinical trials to examine the benefit of myosin inhibitors in non-obstructive HCM with data likely expected in early 2025.^40^ There are also efforts to determine whether the success of sodium-glucose cotransporter-2 inhibitors seen in heart failure with preserved ejection fraction may also be of benefit in non-obstructive HCM.^41^

### Limitations

There are limitations to this study. Only English language guidelines were considered, which may lead to an incomplete view of the evidence base and introducing selection bias. This was mitigated against by using a comprehensive search strategy as well as manual searching of national and international cardiovascular societal websites and the selection process being performed independently by two reviewers. This review is limited to recommendations for adult patients. It was beyond the scope of this paper to evaluate the strengths of the individual guideline recommendations.

## Conclusions

Contemporary guidelines for HCM achieve consensus across a broad range of important criteria concerning diagnosis and management. There are differences in the approach towards risk assessment for SCD and the utility of LGE imaging, which will likely evolve as the evidence-base broadens.

## Supplementary Material

qcae117_Supplemental_Files
