# Supplementary material for: Hypertrophic cardiomyopathy management: a systematic review of the clinical practice guidelines and recommendations
Source: Eur Heart J Qual Care Clin Outcomes. 2025 Jan 2;11(7):919–33. doi: 10.1093/ehjqcco/qcae117 (PMC12587277; doi:10.1093/ehjqcco/qcae117)
Supplement: qcae117_Supplemental_Files [file qcae117_supplemental_files.zip › AGREE scores.docx]

**Supplementary Online Material**

**Supplementary Table 1.**

**Website searches of guideline development organizations, including websites**

| **Organization Responsible for Guideline Development** | **Country** | **Website Searched** |
| --- | --- | --- |
| American Academy of Family Physicians | United States | www.aafp.org |
| American Association of Thoracic Surgery | United States | www.aats.org |
| American College of Cardiology | United States | www.acc.org |
| American College of Physicians | United States | www.acponline.org |
| American College of Surgeons | United States | www.facs.org |
| American College for Preventive Medicine | United States | www.acpm.org |
| American College of Radiology | United States | www.acr.org |
| American Geriatrics Society | United States | www.americangeriatrics.org |
| American Heart Association | United States | www.americanheart.org |
| American Society of Echocardiography | United States | www.asecho.org |
| American Society of Nuclear Cardiology | United States | www.asnc.org |
| American Medical Association | United States | www.ama-assn.org |
| Australian Medical Association | Australia | www.ama.com.au |
| British Cardiovascular Society | United Kingdom | www.bcs.com |
| British Society of Echocardiography | United Kingdom | www.bsecho.org |
| British Society for Heart Failure | United Kingdom | www.bsh.org.uk |
| Canadian Cardiovascular Society | Canada | www.ccs.ca |
| Canadian Heart Failure Society | Canada | www.heartfailure.ca |
| Canadian Task Force on Preventive Health Care | Canada | www.canadiantaskforce.ca |
| Cardiac Society of Australia and New Zealand | Australia | www.csanz.edu.au |
| Centers for Disease Control and Prevention/American Heart Association | United States | www.cdc.gov |
| Department of Health | United Kingdom | www.dh.gov.uk/en |
| European Association of Cardiovascular Imaging | Europe | www.escardio.org/Sub-specialty-communities/European-Association-of-Cardiovascular-Imaging-(EACVI) |
| European Association for Cardio-Thoracic Surgery | Europe | www.eacts.org |
| European Society of Cardiology | Europe | www.escardio.org |
| Heart Failure Association of the ESC | Europe | www.escardio.org/Sub-specialty-communities/Heart-Failure-Association-of-the-ESC-(HFA) |
| Heart Failure Society of America | United States | www.hfsa.org |
| Heart Rhythm Society | United States | www.hrsonline.org |
| Heart Valve Society | United States | www.heartvalvesociety.org |
| Japanese Circulation Society | Japan | www.j-circ.or.jp |
| Japanese Heart Failure Society | Japan | www.asas.or.jp/jhfs |
| Japanese Society of Echocardiography | Japan | www.jse.gr.jp |
| Japanese Society of Ultrasonic in Medicine | Japan | www.jsum.or.jp |
| National Health and Medical Research Council | Australia | www.nhmrc.gov.au |
| National Heart Foundation | Australia | www.heartfoundation.org.au |
| National Heart Lung and Blood Institute | United States | www.nhlbi.nih.gov/guidelines |
| National Institute for Health and Care Excellence | United Kingdom | www.nice.org.uk |
| New Zealand Guidelines Group | New Zealand | www.nzgg.org.nz |
| Royal College of General Practitioners | United Kingdom | www.rcgp.org.uk |
| Scottish Intercollegiate Guidelines Network | United Kingdom | www.sign.ac.uk |
| Society of Cardiovascular Computed Tomography | United States | www.scct.org |
| Society for Cardiovascular Magnetic Resonance | United States | www.scmr.org |
| Society of Critical Care Medicine | United States | www.sccm.org |
| The Society for Cardiovascular Angiography and Interventions | United States | www.SCAI.org |
| The Society of Thoracic Surgeons | United States | www.sts.org |
| U.S. Preventive Services Task Force | United States | www.ahrq.gov |
| World Heart Federation | International | www.world-heart-federation.org |
| World Health Organization | International | www.who.int |

| **AGREE Scoring** |  |  |  | |  | |  |  |  |  |  |  |
| --- | --- | --- | --- | --- | --- | --- | --- | --- | --- | --- | --- | --- |
| **Guideline** | Reviewer | Method to search for evidence | Criteria to select evidence | | Strengths and limitations of evidence | | Methods for formulating recommendations | Health benefits, side effects, and risks | Link between recommendations and evidence | Procedures for external expert review | Updating process | Domain score, % |
|  |  |  | |  | |  |  |  |  |  |  |  |
| **AHA/ACC/AMSSM/HRS/PACES/SCMR** | A | 7 | | 4 | | 7 | 3 | 7 | 7 | 6 | 5 | 82% |
|  | B | 6 | | 4 | | 6 | 4 | 7 | 7 | 5 | 5 | 80% |
| **ESC** | A | 2 | | 2 | | 6 | 5 | 7 | 6 | 6 | 3 | 66% |
|  | B | 3 | | 3 | | 6 | 4 | 6 | 7 | 5 | 4 | 60% |
| **JCS/JHFS** | A | 1 | | 2 | | 5 | 1 | 7 | 6 | 2 | 4 | 50% |
|  | B | 2 | | 3 | | 4 | 2 | 6 | 5 | 3 | 4 | 52% |

**Search Strategy**

Embase <1974 to 2024 May 20>

Ovid MEDLINE(R) ALL <1946 to May 20, 2024>

1 "Cardiomyopathy, Hypertrophic"/ 39919

2 "Cardiomyopathy, Hypertrophic, Familial"/ 1233

3 "Cardiomyopathies"/ 73013

4 "cardiomyopathy".ab,ti. 224836

5 "cardiomyopathies".ab,ti. 17398

6 "hypertrophic cardiomyopathy".ab,ti. 43428

7 "HCM".ab,ti. 20691

8 "idiopathic hypertrophic subaortic stenosis".ab,ti. 853

9 "asymmetric septal hypertrophy".ab,ti. 1201

10 "familial hypertrophic cardiomyopathy".ab,ti. 1596

11 "HOCM".ab,ti. 2852

12 "hypertrophic obstructive cardiomyopathy".ab,ti. 5543

13 "apical hypertrophic cardiomyopathy".ab,ti. 1508

14 "apical HCM".ab,ti. 406

15 "hypertrophic non-obstructive cardiomyopathy".ab,ti. 161

16 1 or 2 or 3 or 4 or 5 or 6 or 7 or 8 or 9 or 10 or 11 or 12 or 13 or 14 or 15 277547

17 (guideline* or "practice guideline" or "consensus development conference" or "consensus development conference, NIH" or guidance* or "position paper" or "position stand" or statement* or recommendation* or consensus or "practice parameter*").pt. 48400

18 (guideline* or standard* or recommend* or "practice parameter*" or "position statement*" or "policy statement*" or CPG* or "best practice*" or guidance* or "position paper" or "position stand" or recommendation* or consensus).ti. 772815

19 (care adj2 (path or paths or pathway or pathways or map or maps or plan or plans or standard)).ti. 23258

20 ((critical or clinical or practice) adj2 (path or paths or pathway or pathways or protocol*)).ti. 11798

21 (guideline* or standards or consensus* or recommendat* or scientifi*).au. 55

22 exp Guideline/ 38464

23 17 or 18 or 19 or 20 or 21 or 22 810981

24 16 and 23 2401

25 limit 24 to yr="2014 -Current" 1617

26 remove duplicates from 25 1145
