# Supplementary material for: Hypertrophic cardiomyopathy management: a systematic review of the clinical practice guidelines and recommendations
Source: Eur Heart J Qual Care Clin Outcomes. 2025 Jan 2;11(7):919–33. doi: 10.1093/ehjqcco/qcae117 (PMC12587277; doi:10.1093/ehjqcco/qcae117)
Supplement: qcae117_Supplemental_Files [file qcae117_supplemental_files.zip › AGREE scores.pdf]

Supplementary Online Material

Supplementary Table 1.

Website searches of guideline development organizations, including websites

Organization Responsible for Guideline Development	Country	Website Searched
American Academy of Family Physicians	United States	www.aafp.org
American Association of Thoracic Surgery	United States	www.aats.org
American College of Cardiology	United States	www.acc.org
American College of Physicians	United States	www.acponline.org
American College of Surgeons	United States	www.facs.org
American College for Preventive Medicine	United States	www.acpm.org
American College of Radiology	United States	www.acr.org
American Geriatrics Society	United States	www.americangeriatrics.org
American Heart Association	United States	www.americanheart.org
American Society of Echocardiography	United States	www.asecho.org
American Society of Nuclear Cardiology	United States	www.asnc.org

American Medical Association	United States	www.ama-assn.org
Australian Medical Association	Australia	www.ama.com.au
British Cardiovascular Society	United Kingdom	www.bcs.com
British Society of Echocardiography	United Kingdom	www.bsecho.org
British Society for Heart Failure	United Kingdom	www.bsh.org.uk
Canadian Cardiovascular Society	Canada	www.ccs.ca
Canadian Heart Failure Society	Canada	www.heartfailure.ca
Canadian Task Force on Preventive Health Care	Canada	www.canadiantaskforce.ca
Cardiac Society of Australia and New Zealand	Australia	www.csanz.edu.au
Centers for Disease Control and Prevention/American Heart Association	United States	www.cdc.gov
Department of Health	United Kingdom	www.dh.gov.uk/en
European Association of Cardiovascular Imaging	Europe	www.escardio.org/Sub-specialty-communities/European-Association-of-Cardiovascular-Imaging-(EACVI)
European Association for Cardio-Thoracic Surgery	Europe	www.eacts.org

European Society of Cardiology	Europe	www.escardio.org
Heart Failure Association of the ESC	Europe	www.escardio.org/Sub-specialty-communities/Heart-Failure-Association-of-the-ESC-(HFA)
Heart Failure Society of America	United States	www.hfsa.org
Heart Rhythm Society	United States	www.hrsonline.org
Heart Valve Society	United States	www.heartvalvesociety.org
Japanese Circulation Society	Japan	www.j-circ.or.jp
Japanese Heart Failure Society	Japan	www.asas.or.jp/jhfs
Japanese Society of Echocardiography	Japan	www.jse.gr.jp
Japanese Society of Ultrasonic in Medicine	Japan	www.jsum.or.jp
National Health and Medical Research Council	Australia	www.nhmrc.gov.au
National Heart Foundation	Australia	www.heartfoundation.org.au
National Heart Lung and Blood Institute	United States	www.nhlbi.nih.gov/guidelines
National Institute for Health and Care Excellence	United Kingdom	www.nice.org.uk
New Zealand Guidelines Group	New Zealand	www.nzgg.org.nz

Royal College of General Practitioners	United Kingdom	www.rcgp.org.uk
Scottish Intercollegiate Guidelines Network	United Kingdom	www.sign.ac.uk
Society of Cardiovascular Computed Tomography	United States	www.scct.org
Society for Cardiovascular Magnetic Resonance	United States	www.scmr.org
Society of Critical Care Medicine	United States	www.sccm.org
The Society for Cardiovascular Angiography and Interventions	United States	www.SCAI.org
The Society of Thoracic Surgeons	United States	www.sts.org
U.S. Preventive Services Task Force	United States	www.ahrq.gov
World Heart Federation	International	www.world-heart-federation.org
World Health Organization	International	www.who.int

AGREE Scoring

**Guideline**	Reviewer	Method to search for evidence	Criteria to select evidence	Strengths and limitations of evidence	Methods for formulating recommendations	Health benefits, side effects, and risks	Link between recommendations and evidence	Procedures for external expert review	Updating process	Domain score, %
**AHA/ACC/AMSSM/HRS/PACES/SCMR**	A	7	4	7	3	7	7	6	5	82%
	B	6	4	6	4	7	7	5	5	80%
**ESC**	A	2	2	6	5	7	6	6	3	66%
	B	3	3	6	4	6	7	5	4	60%
**JCS/JHFS**	A	1	2	5	1	7	6	2	4	50%
	B	2	3	4	2	6	5	3	4	52%

Search Strategy

Embase <1974 to 2024 May 20>
Ovid MEDLINE(R) ALL <1946 to May 20, 2024>

1 "Cardiomyopathy, Hypertrophic"/ 39919
2 "Cardiomyopathy, Hypertrophic, Familial"/ 1233
3 "Cardiomyopathies"/ 73013
4 "cardiomyopathy".ab,ti. 224836
5 "cardiomyopathies".ab,ti. 17398
6 "hypertrophic cardiomyopathy".ab,ti. 43428
7 "HCM".ab,ti. 20691
8 "idiopathic hypertrophic subaortic stenosis".ab,ti. 853
9 "asymmetric septal hypertrophy".ab,ti. 1201
10 "familial hypertrophic cardiomyopathy".ab,ti. 1596
11 "HOCM".ab,ti. 2852
12 "hypertrophic obstructive cardiomyopathy".ab,ti. 5543
13 "apical hypertrophic cardiomyopathy".ab,ti. 1508
14 "apical HCM".ab,ti. 406
15 "hypertrophic non-obstructive cardiomyopathy".ab,ti. 161
16 1 or 2 or 3 or 4 or 5 or 6 or 7 or 8 or 9 or 10 or 11 or 12 or 13 or 14 or 15 277547
17 (guideline* or "practice guideline" or "consensus development conference" or "consensus development conference, NIH" or guidance* or "position paper" or "position stand" or statement* or recommendation* or consensus or "practice parameter*").pt. 48400
18 (guideline* or standard* or recommend* or "practice parameter*" or "position statement*" or "policy statement*" or CPG* or "best practice*" or guidance* or "position paper" or "position stand" or recommendation* or consensus).ti. 772815
19 (care adj2 (path or paths or pathway or pathways or map or maps or plan or plans or standard)).ti. 23258
20 ((critical or clinical or practice) adj2 (path or paths or pathway or pathways or protocol*)).ti. 11798
21 (guideline* or standards or consensus* or recommendat* or scientifi*).au. 55
22 exp Guideline/38464
23 17 or 18 or 19 or 20 or 21 or 22 810981
24 16 and 23 2401
25 limit 24 to yr="2014 -Current" 1617
26 remove duplicates from 25 1145
